# First report of rice root-knot nematode, *Meloidogyne graminicola*, infecting *Juncus microcephalus* in Brazil

**DOI:** 10.21307/jofnem-2021-031

**Published:** 2021-03-20

**Authors:** Cristiano Bellé, Paulo Sergio dos Santos, Tiago Edu Kaspary

**Affiliations:** 1 Phytus Group, Estação Experimental de Itarra, Estrada da Estação, 3219, Interior, 97185-000, Itaara, RS, Brazil; 2 Phytus Group, Estação Experimental de Planaltina, Rodovia DF, no. 145, Km 3, 73301-970, Planaltina, DF, Brazil; Instituto Nacional de Investigación Agropecuaria – INIA La Estanzuela, Colonia, Uruguay

**Keywords:** Identification, Molecular analyses, EST phenotype, Root-knot nematodes

## Abstract

*Juncus microcephalus* plants showing symptoms of root-knot nematode infestation were observed in the municipality of Agudo, Rio Grande do Sul state, Brazil. Based on morphological observation, esterase phenotypes, and molecular analyses of rDNA-ITS and D2-D3 regions of 28S rDNA, the causal agent of the observed symptoms was identified as *Meloidogyne graminicola*. Pathogenicity of *M. graminicola* was confirmed by fulfilling modified Koch’s postulates. To our knowledge, this is the first report of *M. graminicola* in *J. microcephalus* in Rio Grande do Sul State, Brazil.

Weeds infest agricultural fields in all productive regions of the world. The presence of these species, infesting commercial crops, reduce potential of productivity by competing for environmental resources, allelopathic effects, and serving as alternative hosts for pests and pathogens (Bellé et al., 2019; Webster and Nichols, 2012). The *Juncus microcephalus* (South American rush) is a weed species originally from south America and is present in wetlands cultivated in many countries worldwide (Balslev and Stefano, 2015; Pivari et al., 2019; Rolon et al., 2010).

*Juncus microcephalus* Kunth (family Juncaceae) is a persistent and herbaceous weed which develops in flooded areas. This weed blooms and fructifies irregularly throughout the year, with greater seed production in the summer (Balslev and Stefano, 2015). South American rush is present in rice fields as weed throughout the development of rice, competing with the crop and reducing its productive potential. In addition, this weed develops during the absence of crop, serving as an alternative host for many crop pests including arthropods, pathogens, including nematodes.

In October 2020, samples of South American reeds showing many galls on the roots (Fig. 1A, B) were collected in rice fields, before sowing the crop from the municipality of Agudo (29° 34′16, 7″S; 53° 17′17, 4″ O; 53 m), state of Rio Grande do Sul, Brazil. No symptoms were observed in the aerial part of infected South American rush plants.

**Figure 1:**
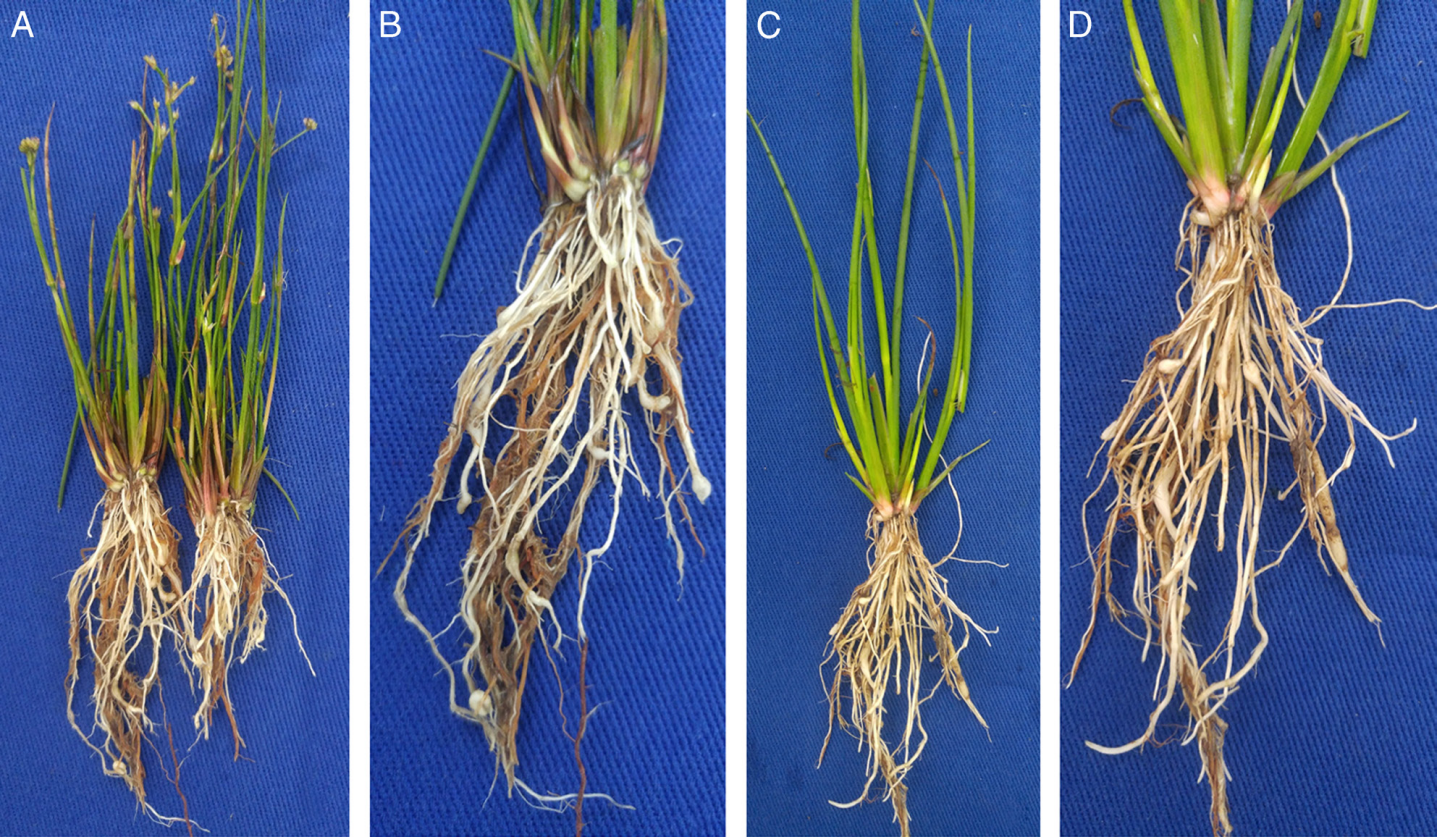
*Meloidogyne graminicola* Golden and Birchfield, 1965, root infestation symptoms on South American rush (*Juncus microcephalus* Kunth). Root-knot symptoms of galls of *J. microcephalus* from the field (A, B) and in the greenhouse (C, D).

This species was identified from esterase using esterase phenotypes (*n* = 20 females) (Carneiro and Almeida 2001; Carneiro et al., 2000), morphological measurement of second-stage juveniles (J2) (*n* = 20), females (*n* = 10) and males (*n* = 10), and perineal patterns (*n* = 20) and through the amplification and sequencing of ITS1-5.8S-ITS2 rRNA region and the D2 to D3 fragment of the 28S ribosomal RNA gene (De Ley et al., 1999; Schmitz et al., 1998). Genomic deoxyribonucleid acid (DNA) was ultimately obtained from females using the NaOH method (Stanton et al., 1998).

The nematode population density observed in the sample was 1,980 J2/g of *J. microcephalus* roots. The J2s had the following morphometric characters: length (L) = 499.5 ± 45.0 (389.0-500.5) μm, *a* = 26.5 ± 1.1 (24.5-30.0), *c* = 5.1 ± 0.4 (4.90-7.1), stylet length =14.9 ± 0.5 (13.3-16.1) μm, dorsal esophageal gland opening (DGO) = 3.6 ± 0.4 (3.1-4.5) μm, tail length = 70.2 ± 3.2 (61.5-79.9) μm and hyaline tail terminus = 18.0 ± 1.3 (15.1-23.5) μm. Morphological measurements of females included *L* = 690.5 ± 30.5 (500.5-799.5) μm, stylet length = 13.5 ± 0.3 (11.2-14.7) μm, and DGO = 3.6 ± 0.4 (3.1-5.5) μm. The female’s perineal patterns were oval shape and a low dorsal arch without the presence of a lateral lines and the cuticular striations were smooth and thick in the dorsal region of the vulva. Male measurements were *L* = 1,401.5 ± 150.5 (1,150.5-1,792.0) μm, stylet length = 19.5 ± 0.5 (17.9-20.2) μm, DGO = 3.5 ± 0.5 (2.4-4.0) μm, tail = 11.4 ± 1.5 (9.4-14.0) μm, spicule = 31.0 ± 1.4 (28.0-36.5) μm. The overall morphology and morphometrics of the population of fit into *Meloidogyne graminicola* (Golden and Birchfield, 1965) according to the original description (Golden and Birchfield, 1965).

The polymorphisms of the esterase bands observed by electrophoresis revealed the phenotype VS-1 (G1) (Rm = 0.70) typical of *M. graminicola* (Carneiro et al., 1996). The sequences of the rDNA regions (ITS: 433 bp and D2-D3 of 28S: 446 bp) were submitted to GenBank (ITS: MW537706 and D2-D3 of 28S: MW537709). Searches on BLAST showed 99 to 100% identity with sequences of *M. graminicola* isolates from Brazil, Taiwan, and China.

To satisfy a modified Koch’s postulates, *J. microcephalus* plantlets were grown in 1.7 L pots filled with a sterilized soil. The seeds were obtained directly from the weeds in the rice fields. Seeds were sown in trays filled with commercial substrate. In total, 20 days after emergence, the seedlings were transplanted to pots (one per pot), five days after transplanting, six plantlets were inoculated with 2,000 eggs and J2s from the original population of *M. graminicola*, extracted with 0.5% NaOCl according to Hussey and Barker (1973), using a blender instead of manual shaking. In addition, non-inoculated control six plants were also included in the study. Plants were maintained under greenhouse conditions at 25 ± 3°C, with watering as needed. After 60 days, the inoculated plants exhibited galled root systems similar observed in the field, with a nematode reproduction factor (final population/initial population) of 9.5 (Fig. 1C, D). Plants did not exhibit any above-ground symptoms. The non-inoculated plants did not exhibit any galls.

Rice plants (cultivars IRGA 424 RI, Guri Inta CL, IRGA 431 CL, and Puita Inta CL) five days after emergence, seedlings were transplanted to 1.7 L pots containing sterilized soil, one plant per pot. Plants were inoculated five days after transplanting were also inoculated with an isolate of *M. graminicola* from *J. microcephalus* using the same methods as described above. The plants exhibited galled root systems with a nematode reproduction factor ranging from 12.4 to 25.5 (Fig. 2A-D). Inoculated plants showed a reduction in fresh weight of shoots (25-29%) and roots (27-30%) compared to non-inoculated plants. These results confirmed the pathogenicity of the *M. graminicola* in *J. microcephalus* and rice.

**Figure 2:**
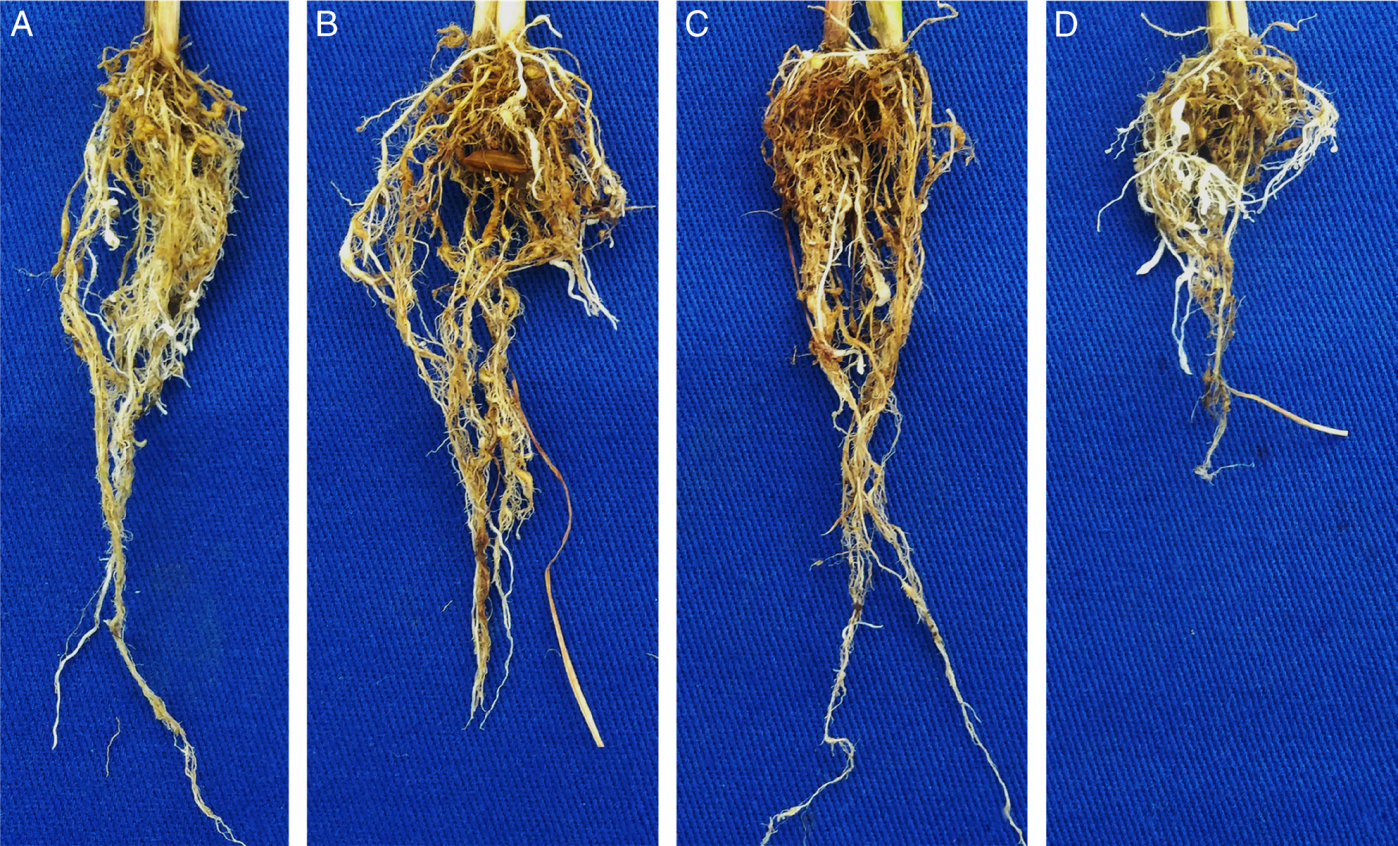
Pathogenicity of *Meloidogyne graminicola* Golden and Birchfield, 1965, isolated from *Juncus microcephalus* Kunth, in different rice cultivars (A: IRGA 424 RI, B: Guri Inta CL, C: IRGA 431 CL and D: Puita Inta CL).

An effective control of nematodes, limiting the increase in the population of these phytoparasites is the proper management of weeds throughout the year including the period of absence of crops where only weeds serves the hosts for this phytoparasite (Bellé et al., 2016). In this sense, the control method most used and considered most efficient for the management of *J. microcephalus* is the chemical with the use of herbicides. In this way, by controlling the weeds, there will be no shelter and food for the nematodes, leading to gradual reduction in their population.

Therefore, the management of weeds is of great importance, since it directly impacts the survival of *Meloidogyne* spp. in agricultural fields, while the negative potential of weeds species on commercial crops is exacerbated when they become phytoparasite hopers. However, based on the knowledge of *M. graminicola* polyphagia and its host range, effective strategies can be devised in the management of this pathogen, reducing the damage caused to commercial crops. Finally, the reduction of agricultural losses caused by weeds and nematodes can be minimized with the integrated management of these two problems, which are interconnected and enhanced when they occur concurrently. To our knowledge, this is the first report of *M. graminicola* parasitizing *J. microcephalus* in Brazil and elsewhere.
